# Dysregulated miRNAs modulate tumor microenvironment associated signaling networks in pancreatic ductal adenocarcinoma

**DOI:** 10.1093/pcmedi/pbad004

**Published:** 2023-03-10

**Authors:** Tiantian Liu, Zhong Chen, Wanqiu Chen, Ryan Evans, Jane Xu, Mark E Reeves, Michael E de Vera, Charles Wang

**Affiliations:** Center for Genomics, School of Medicine, Loma Linda University, Loma Linda, CA 92350, USA; Center for Genomics, School of Medicine, Loma Linda University, Loma Linda, CA 92350, USA; Center for Genomics, School of Medicine, Loma Linda University, Loma Linda, CA 92350, USA; Transplant Institute, Loma Linda University, Loma Linda, CA 92350, USA; Center for Genomics, School of Medicine, Loma Linda University, Loma Linda, CA 92350, USA; Cancer Center & School of Medicine, Loma Linda University, Loma Linda, CA 92350, USA; Transplant Institute, Loma Linda University, Loma Linda, CA 92350, USA; Center for Genomics, School of Medicine, Loma Linda University, Loma Linda, CA 92350, USA; Department of Basic Sciences, School of Medicine, Loma Linda University, Loma Linda, CA 92350, USA

**Keywords:** pancreatic cancer, RNA-seq, miRNA-seq, tumor microenvironment, single-cell RNA-sequencing

## Abstract

The desmoplastic and complex tumor microenvironment of pancreatic ductal adenocarcinoma (PDAC) has presented tremendous challenges for developing effective therapeutic strategies. Strategies targeting tumor stroma, albeit with great potential, have met with limited success due to the lack of knowledge on the molecular dynamics within the tumor microenvironment (TME). In pursuit of a better understanding of the influence of miRNAs on TME reprogramming and to explore circulating miRNAs as diagnostic and prognostic biomarkers for PDAC, using RNA-seq, miRNA-seq, and single-cell RNA-seq (scRNA-seq), we investigated the dysregulated signaling pathways in PDAC TME modulated by miRNAs from plasma and tumor tissue. Our bulk RNA-seq in PDAC tumor tissue identified 1445 significantly differentially expressed genes with extracellular matrix and structure organization as the top enriched pathways. Our miRNA-seq identified 322 and 49 abnormally expressed miRNAs in PDAC patient plasma and tumor tissue, respectively. We found many of the TME signaling pathways were targeted by those dysregulated miRNAs in PDAC plasma. Combined with scRNA-seq from patient PDAC tumor, our results revealed that these dysregulated miRNAs were closely associated with extracellular matrix (ECM) remodeling, cell-ECM communication, epithelial-mesenchymal transition, as well as immunosuppression orchestrated by different cellular components of TME. The findings of this study could assist the development of miRNA-based stromal targeting biomarkers or therapy for PDAC patients.

## Teaser

This study revealed that miRNAs were involved in regulating extracellular matrix (ECM) remodeling, cell-ECM communication, epithelial-mesenchymal transition, as well as immunosuppression orchestrated by different cellular components of TME. The findings could assist the development of miRNA based stromal targeting biomarkers or therapy for PDAC patients.

## Background

Pancreatic cancer is one of the most lethal malignancies and the third leading cause of cancer related death in the U.S., according to American Cancer Society.^1^ Pancreatic ductal adenocarcinoma (PDAC) represents the majority of the incidence. The desmoplastic and immunosuppressive tumor microenvironment (TME) is one of crucial hallmarks of PDAC.^1,2^ Comprised of all non-malignant cellular and non-cellular components of the tumor, the TME includes immune cells, fibroblasts, endoblasts, extracellular matrix (ECM), as well as all the signaling molecules in the tumor niche.^2^ During PDAC progression, cancer cells and stroma interaction promote the reprogramming of ECM and create a fibrotic, hypoxic, and mostly immunosuppressive environment which favors tumor growth and renders drug resistance,^1,3,4^ albeit tumor-suppressive cells were also found in TME.^5^ Using microarray and RNA-seq technologies, researchers have revealed a distinct mitotic expression signature in PDAC TME, which was associated with the progression and metastasis. However, those most differentially expressed genes in PDAC TME were linked to and primarily separated by tissue compartments, but not in a tumor specific manner, making it difficult to choose specific genes or gene sets as PDAC biomarkers.^6^ Other attempts to find PDAC TME biomarkers for diagnostic and therapeutic purpose have also been disappointing, due to the complex, multi-faceted roles of stroma and the stromal heterogeneity between and within patients.^5^ In addition, PADC tissue level biomarker searching requires time-consuming biopsy or surgery procedure. Therefore, the development of noninvasive biomarker from patient blood would be more favorable and benefit diagnostic and therapeutic strategy.^5,7^

MicroRNAs (miRNAs) are small non-coding RNAs that regulate gene expression by inducing mRNA degradation and inhibiting translation.^8^ Abnormal miRNA expression has been reported in several malignant tumors, including PDAC, and has been closely linked to tumor progression and chemoresistance.^9,10^ Recent studies pointed out that miRNAs regulate TME remodeling by facilitating tumor-stroma communication. This is achieved by inhibiting their target genes and consequently activating cancer associated fibroblast (CAFs), tumor-associated macrophages (TAMs), as well as tumor infiltrating lymphocytes.^11^ Furthermore, studies have suggested that tumor-derived exosomal miRNAs are key regulators for TME heterogeneity by acting as chemical messengers between cancer cells and stromal cells, leading to tumor progression.^12^ Therefore, examining TME signaling modulated by miRNAs in PDAC is crucial for understanding the structural and functional dynamics of the PDAC TME and will aid the development of stroma-targeted therapeutic strategies against PDAC.

Multiple studies have explored miRNAs, especially circulating miRNAs, as potential prognostic and/or diagnostic biomarkers for PDAC.^9^ However, studies on miRNAs, especially tumor-derived circulating miRNAs as PDAC TME or tumor stroma biomarkers are still scarce. By integrating RNA-seq and miRNA-seq, we sought to identify some dysregulated miRNAs as potential PDAC TME biomarkers. We further carried out single-cell RNA-seq^13^ on PDAC tumor to characterize the dynamics and specificity of miRNA modulation on TME signaling in the heterogeneous PDAC tumor context at single-cell resolution. Our results not only shed light on the miRNA-mediated PDAC TME remodeling, but also presented evidence to use dysregulated miRNAs as potential biomarkers for PDAC prognosis and progression assessment.

## Methods

### Tissue specimen and plasma collection

The study was approved by the Institutional Review Board of the Loma Linda University (LLU) and informed consents were obtained from all subjects. All the clinical specimens were collected at LLU Medical Center according to IRB approved protocol. Six chemotherapy naïve PDAC patients were recruited for this study (Supplementary Table S1). Fresh tumor and adjacent peri-tumoral tissue were obtained during surgical resections from PDAC patients. For bulk level sequencing, tissues were stored in -80°C before RNA extraction. For single cell sequencing, tissue was processed immediately. For each PDAC patient 8 ml of peripheral blood was drawn into BD Vacutainer Mononuclear Cell Preparation Tubes (CPT) (Becton, Dickinson and Company, Franklin Lakes, NJ) with sodium citrate during the surgery. The CPTs were processed following the manufacturer's protocol. For each sample, 2 ml of plasma was collected from the upper clear layer after centrifugation of CPTs at 1300x g for 30 minutes at room temperature. The isolated plasma was further spun at 12 000x g and 4°C for 15 minutes to remove cells and debris.

### Tissue bulk-cell RNA sequencing

Total RNA was extracted from five PDAC subjects’ tumor and related four peri-tumor frozen tissues using miRNeasy kit (Qiagen, Redwood City, CA). Bulk RNA-seq libraries were constructed using Tecan Universal RNA-seq Library Preparation kit (Tecan, San Jose, CA). Briefly, RNAs were reversely transcribed to generate double strand cDNAs. After end repair, adaptors with unique barcode were ligated to cDNAs. AnyDeplete probes for human were used to deplete rRNA. RNA-seq libraries were amplified and purified using Agencourt AMPure XP Beads (Beckman Coulter, Brea, CA). Library quality was examined using the TapeStation 2200 (Agilent, Santa Clara, CA). Libraries were quantified using Qubit 3.0 HS dsDNA assay (Thermal Fisher Scientific, Waltham, MA). The RNA-seq libraries were pooled and sequenced as pair-end (80 bpx2) on an Illumina HiSeq 4000 (Illumina, San Diego, CA) at the Loma Linda University Center for Genomics.

### miRNA sequencing

Circulating RNA were extracted from 200 *μ*l of plasma using miRNeasy Serum/Plasma Kit (Qiagen, Redwood City, CA) according to manufacturer's protocol. Five PDAC patients and six healthy control subjects were included in plasma miRNA study; while five PDAC subjects’ tumor and the related four peri-tumoral tissue samples were used in tissue miRNA study. miRNA-seq libraries were constructed from both tissue total RNA and circulating miRNA using QIAseq miRNA library kit (Qiagen, Redwood City, CA). Briefly, after 3′ and 5′ adaptor ligation, the target miRNAs were reversely transcribed into cDNAs and a unique molecular index (UMI) was assigned to each miRNA molecule. After cDNA cleanup, the miRNA-seq libraries were amplified by 22-cycles of PCR during which a unique sample index was added to each sample. The amplified DNA fragments were subject to double size selection using QIAseq magnetic beads to select the molecules with sizes ranging from 150–200 bp. The libraries were quantified using Qubit 3.0 HS dsDNA assay, and the library quality was examined using D1000 Screentape on TapeStation 2200. The miRNA-seq libraries were sequenced at 84 bp single-end reads on an Illumina NextSeq 550 (Illumina, San Diego, CA).

### Single-cell RNA sequencing

Freshly resected tissue was dissociated using Miltenyi tumor dissociation human kit (Miltenyi Biotec, Bergisch Gladbach, Germany) following manufacturer's protocol. GentleMACS™ Dissociator (Miltenyi Biotec, San Jose, CA) was used for enzymatic and mechanic coupled dissociation. After dissociation, cell suspensions were filtered through 70 *μ*m strainers. Red blood cells were removed using Erythrocyte lysis buffer (Qiagen, Redwood City, CA). The number and viability of single cells were assessed using BioRad Cell counter. The dissociated tumor single cells were suspended at 1000 cells/*μ*l in PBS and loaded into 10x Chromium Controller (10x Genomics, Pleasanton, CA). Due to the difficulty of PDAC tumor dissociation, only one PDAC tumor sample (HP01) yielded enough live cells for single-cell RNA sequencing. The single cell capturing, barcoding, and cDNA library were obtained using Chromium Single Cell 5′ Library & Gel bead kit (v1), following manufacturer's protocol. The final library was analyzed on TapeStation 2200 for peak size, quantified by Qubit 3.0 with HS dsDNA reagent, and sequenced on an Illumina NextSeq 550 in pair-ended read (26 + 98 bp).

### Bulk-cell RNA-seq data analysis

All sequencing data were demultiplexed and converted into fastq files by Illumina bcl2fastq (v2.20). Reads quality was assessed using FastQC (v0.11.4). Low quality reads were trimmed and filtered by TrimGalore (v0.4.1). Filtered sequence reads were mapped to reference genome GRCh38 using STAR (v2.6.0) aligner with default settings. The aligned bam files were processed by HTSeq (v0.6.1) for gene quantification. Differentially expressed genes (DEGs) were identified by R package ‘DESeq2’ (v 3.24.3) and only the genes with at least 10 reads in all samples were included in the analysis. The adjacent peri-tumor tissue was used as control to identify DEGs in tumor tissue. DESeq2 performed an internal normalization where geometric mean was calculated for each gene across all samples. The counts for a gene in each sample was then divided by this mean. This procedure could correct for library size and RNA composition bias. DESeq2 fits negative binomial generalized linear models for each gene and uses the Wald test for significance testing. Genes with false discovery rate (FDR) less than 0.05 and absolute log2FoldChange above 1 were considered significant DEGs. Gene Set Enrichment Analysis (GSEA) and Gene Ontology (GO) term enrichment were performed using R package ‘ClusterProfiler’ (v4.4.4). Sample distance heatmap was generated using R package ‘pheatmap’ (v1.0.12). Volcano plot was generated using R package ‘ggplot2’ (3.3.6).

### miRNA-seq data analysis

All sequencing data were demultiplexed and converted into fastq files by Illumina bcl2fastq (v2.20). The reads were mapped and counted using the primary analysis procedure of Qiagen online miRNA-seq analysis software (https://geneglobe.qiagen.com/sg/analyze). Briefly, reads were trimmed off 3′ adaptor and the low-quality bases by Cutadapt and those shorter than 16 bp and less than 10 UMI counts were excluded in the analysis. Alignment was performed using Bowtie with maximum two mismatches. Aligned reads were annotated using miRBase (v21). After removal of duplicates, UMI counts with more than 20 reads were used for miRNA differential expression analysis using R package ‘DESeq2’ (v1.34.0). The adjacent peri-tumor tissue and plasma from healthy subjects were used as control groups to identify differentially expressed miRNAs in tumor tissue and patient plasma, respectively. Same methods were used for normalization and significance test as described in bulk RNA-seq data analysis section. The Pearson correlation plot of miRNA-seq samples was generated using R package ‘corrplot’ (v0.92).

### miRNA-gene integration analysis

Ingenuity Pathway Analysis (IPA) was used for miRNA and target gene integration analysis. Briefly, the target mRNAs of differentially expressed miRNAs (DE miRNAs) were predicted using multiple target prediction tools (TargetScan, miRecord, and TarBase) against either experimentally observed dataset or predicted dataset, respectively. The confidence level was set to high (the cumulative weighted context score, “CWCS”, was -0.4 or lower) when predicted dataset was used for target scanning. The predicted target genes were further identified from the bulk RNA-seq DEGs. Opposite expression pairing, *i.e.*, miRNA expression was inversely correlated to that of its respective target gene expression, was applied to select miRNA-gene pairs. We further performed co-expression analysis on the expressions of tissue DEmiRNAs and their target genes, using Pearson correlation. The correlation plot was generated using R package ‘corrplot’ (v0.92). Since there was no tissue level gene expression data from plasma miRNA from healthy control subjects, co-expression analysis on differential plasma miRNAs and their target genes could not be carried out. miRNA-gene pairs involved in TME signaling pathways were filtered via IPA and the molecular network was generated using IPA pathway designer. Circos plots featuring DEmiRNAs and DEGs were generated using R package ‘RCircos’ (v1.2.1). Kaplan-Meier plots were generated using the Kaplan-Meier plotter (www.kmplot.com), sourcing gene expression and overall survival data from 177 PDAC patients from TCGA.

### Single-cell RNA-seq data analysis

Single-cell sequencing data was processed using Cell Ranger (v5.0.1, 10x Genomics) pipelines for mapping and counting. Briefly, the raw reads were aligned against human reference genome (GRCh38) and the barcodes and UMIs were counted to generate feature-barcode matrix. Downstream analysis and visualization were performed using R package ‘Seurat’ (v3.0). Cells with fewer than 300 genes or 2,000 features detected, or with more than 10% mitochondrial genes were excluded from the downstream analyses. After data normalization and scaling, PCA was performed on the 2,000 most variable genes. Dimensions 1 to 10 were used for Uniform Manifold Approximation and Projection (UMAP) dimension reduction and cell clustering. Gene marker feature plots were generated using FeaturePlot function. Heatmap for the top 10 variable genes by absolute fold-change was generated using DoHeatmap function. Inferred copy number variation in cancer cells was performed using R package ‘inferCNV’ (v1.12.0) with cancer associated fibroblasts (CAFs) as reference cells. To assess the Pearson correlation in gene expression between single-cell RNA-seq and bulk-cell RNA-seq, we summed the gene counts from each single cell for a pooled expression count and calculated the mean gene counts for the bulk-cell RNA-seq tumor and normal group, respectively. The pooled single-cell gene counts and the mean gene counts from bulk-cell RNA-seq were combined to generate a new gene expression count matrix which was normalized by regularized log (rlog) transformation. Pearson correlation coefficients were calculated between the single cell samples and bulk tumor or normal RNA-seq samples, respectively.

## Results

### Overall study design, data (miRNA-seq, bulk-cell RNA-seq, scRNA-seq) generation and QC

**Figure 1.**
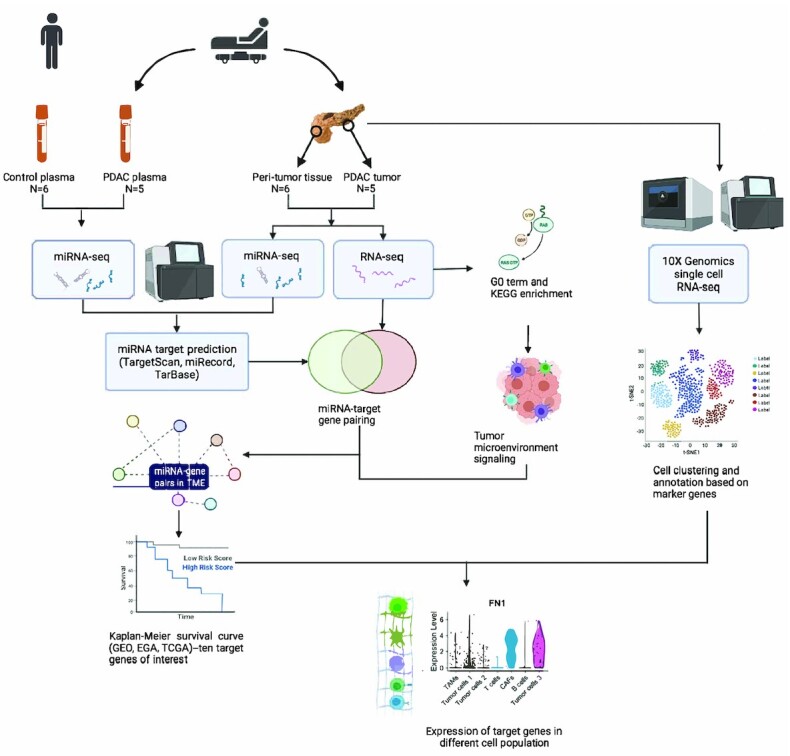
Overall study design.

Overall study design is shown in Fig. 1. Briefly, RNA-seq and miRNA-seq were performed on RNA from the tumor and the peri-tumor "normal" tissues, respectively. Additional miRNA-seq was performed on circulating miRNAs obtained from PDAC patients and healthy subjects. Tumor tissues and adjacent peri-tumor pancreatic tissues were collected from five PDAC patients (HP01, HP02, HP03, HP04 and HP05). As no sufficient amount of adjacent tissue was obtained from HP02 tumor section, only four peri-tumor tissues were used in subsequent RNA-seq analysis. Plasmas from five PDAC patients and six healthy subjects were used in miRNA-seq analysis. miRNA-gene opposite expression analysis was performed to identify gene-miRNA interaction pairs in both tissue and plasma. GO term and GSEA were performed on DEGs as well as DE miRNAs. After identifying miRNA-gene pairs involved in tumor microenvironment signaling, Kaplan-Meier survival analysis was performed to examine the prognostic value for candidate biomarkers. Single-cell RNA-seq was performed on PDAC tumor tissue from subject HP01 to investigate the cell types targeted by these gene-miRNA pairs.

### Extracellular matrix remodeling in PDAC

**Figure 2.**
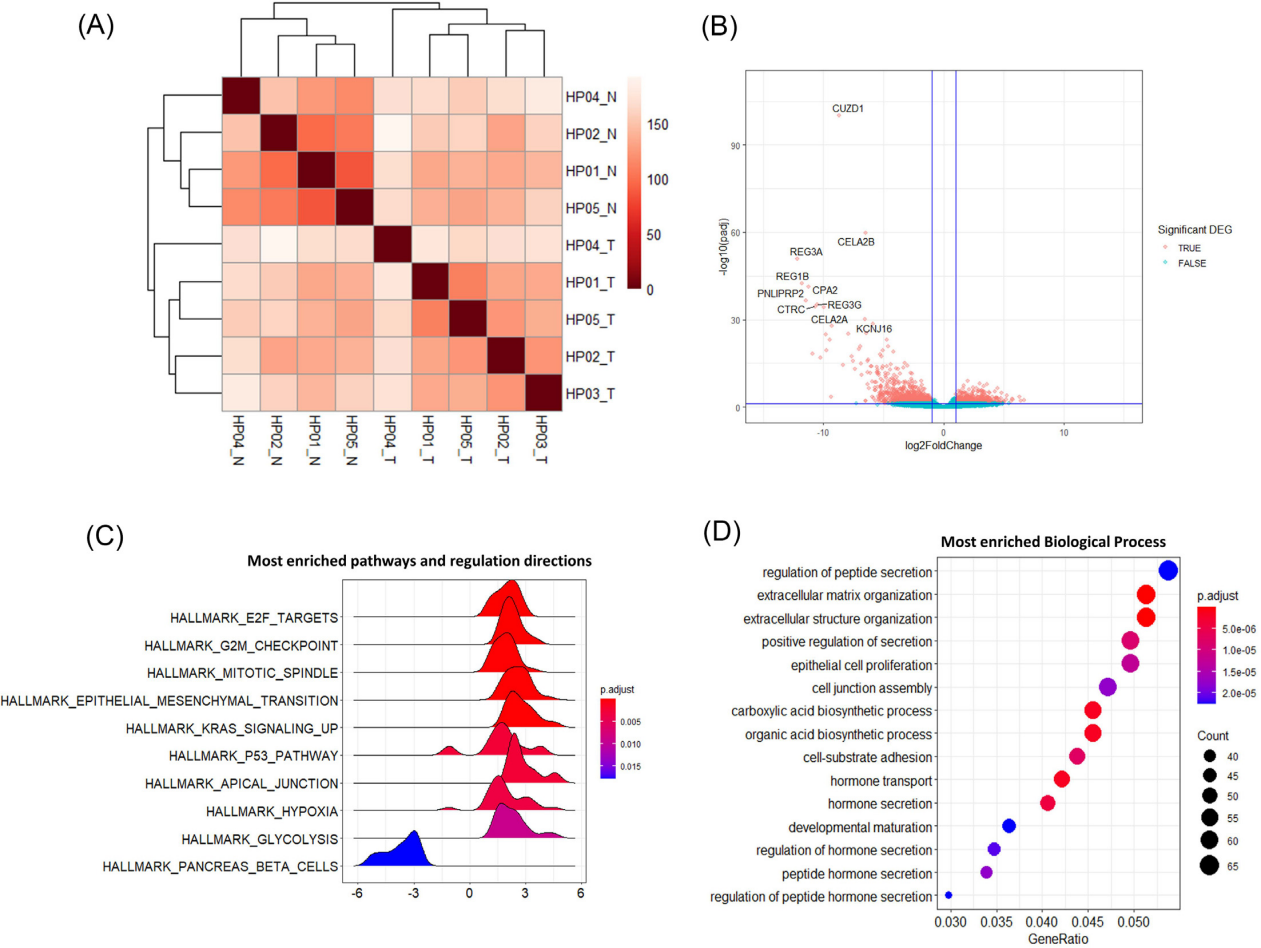
Bulk-cell RNA-seq of PDAC tumor vs. adjacent peri-tumor tissue showing an extracellular matrix remodeling gene expression signature. (**A**) Heatmap of sample distance of 5 PDAC tumoral tissue samples vs. 4 peri-tumor tissue samples. (e.g., HP01_T: HP01 subject, tumor tissue; HP01_N: Subject HP01, peri-tumor tissue). The heatmap was constructed using Deseq2 rlog transformed gene read counts. (**B**) Volcano plot showing the DEGs in PDAC tumor tissue compared to peri-tumor tissue. –log10 (padj) >1.3 (padj < 0.05) and absolute log2FoldChange > 1 were used as the significant thresholds. The top 10 significant DEGs are highlighted. (**C**) Top enriched hallmark pathways by Gene Set Enrichment Analysis (GSEA) based on DEGs identified in PDAC tumor. X-axis indicates the log2FoldChange of each gene enriched in the gene set. (**D**) Top enriched Gene Ontology Biological Processes based on DEGs.

We calculated the sample distance between the five PDAC tumors and four peri-tumor “normal” tissues, based on the normalized gene expression count. As expected, higher similarity in gene expression was found among tumor samples than that between individual patient's tumor and peri-tumor tissue (Fig. 2A). Specifically, we detected 22 062 genes expressed across PDAC tumor and peri-tumor normal tissues in bulk-cell RNA-seq and identified 1445 genes as significant DEGs with FDR < 0.05 and absolute log2FoldChange > 1 (all the DEGs are listed in Supplementary Table S2). Figure 2B shows all the DEGs in a volcano plot. Among the top DEGs, pancreatic lipase-related genes and regenerating family genes were mostly down-regulated in PDAC tumor tissues. Genes encoding proteases for extracellular matrix degradation, like matrix metalloprotease family members (*MMP9* and *MMP13*, log2FoldChange = 6.32 and 4.64, respectively)^11^ and *KLK10* (log2FoldChange = 4.74),^12^ were among the top upregulated genes in the PDAC tumor tissues. In addition, *KRT13*, a gene involved in epithelial-mesenchymal transition (EMT),^14^ was also significantly upregulated in PDAC tumor tissues (log2FoldChange = 5.03). GSEA suggested that KRAS signaling and P53 signaling, two of the PDAC signature pathways, were the most activated pathways in PDAC tumor along with other cancer promoting pathways (Fig. 2C). We also observed the activation of TME reprogramming associated pathways such as hypoxia signaling and epithelial mesenchymal transition (EMT) signaling which indicated a prominent TME network (Fig. 2C). The GO term enrichment identified several enriched Biological Processes, among which the regulation of peptide secretion and extracellular matrix structure organization were the most significantly enriched (Fig.   2D).

### PDAC tumor tissue miRNAs mainly targeted genes in pancreatic secretion

**Figure 3.**
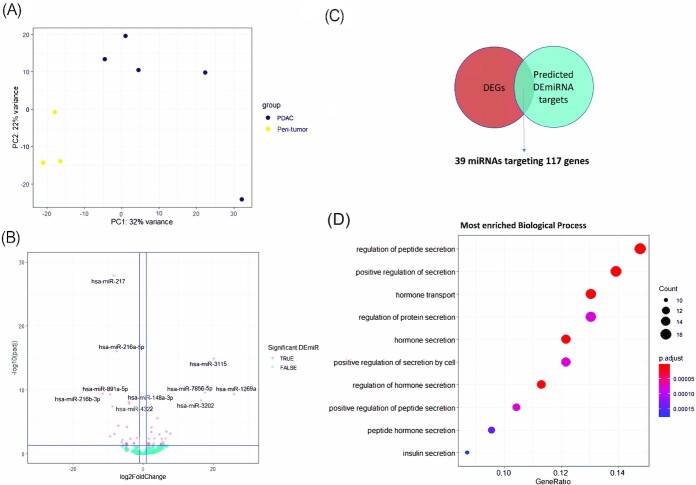
miRNA expression and function analysis in PDAC tumor tissue. (**A**) PCA plot of 5 PDAC tumor tissue samples vs. 3 peri-tumor tissue samples. HP03_N was excluded from the subsequent analysis due to abnormal QC results in miRNA-seq library. (**B**) Volcano plot showing differentially expressed miRNAs between PDAC tumor and peri-tumor tissue. –log10 (padj) >1.3 (padj < 0.05) and absolute log2FoldChange > 1 are marked as the significant threshold. The top 10 significant DE miRNAs are highlighted. (**C**) Venn Diagram showing that 117 of all the DEGs from RNA-seq data were predicated targets of 39 tissue DEmiRNAs. (**D**) Top GO term Biological Process enrichment based on tissue DEmiRNA targeted DEGs.

Besides the RNA-seq data obtained from PDAC tumor tissues, the miRNA expression data was also generated from PDAC tumor tissues and its adjacent peri-tumor tissues, respectively. An average of 5.5 million of reads per sample were mapped to miRNA reference genome. The Principal Component Analysis (PCA) based on global miRNA expression data revealed that PDAC tumor samples were separated from the peri-tumor tissue samples at both PC1 and PC2 (except for a likely outliner at PC2 level in tumor tissue samples), which suggested certain intrinsic heterogeneity on miRNA expression in the PDAC tumor (Fig. 3A). Forty-nine significant DE miRNAs were identified between PDAC tumor and peri-tumor tissue as displayed in the volcano plot (Fig. 3B) and are listed in Supplementary Table S3 in detail. miR-1269a, miR-3115, and miR-3202 were among the most upregulated miRNAs in the PDAC tumor tissues compared to peri-tumor tissues. On the other hand, miR-216 family, including both guide and passenger strands of miR-216b and miR-216a, was among the most downregulated miRNAs in PDAC tissues. It is worth noting that multiple members of miR-216 family were suppressed in PDAC tissues in multiple studies and their anti-tumor roles in PDAC development were investigated by Yonemori *et al*.^15–17^ We identified 143 miRNA-gene interactions (between 39 DEmiRNAs and 117 target DEGs) with high confidence, through IPA pairing analysis between DE miRNAs and their predicted or experimentally verified target genes (Fig. 3C, Supplementary Table S4). To further examine the regulatory role of miRNA on the expression of its target genes, we performed co-expression analysis on all the 143 miRNA-gene pairs (Supplementary Table S5). Overall 83% of those miRNA-gene pairs showed a negative correlation, while 12% of those pairs showed strong negative correlation (Supplementary Fig. S6). This data clearly supported the view that PDAC tumor tissue miRNAs play a key role in regulating tumor gene expression. The GO term enrichment based on the target DEGs indicated that these DEmiRNAs in PDAC tumor tissues mostly target the pancreatic functions such as hormone secretion and transportation (Fig. 3D).

### Circulating miRNAs regulated PDAC TME remodeling and their potential as PDAC biomarkers

**Figure 4.**
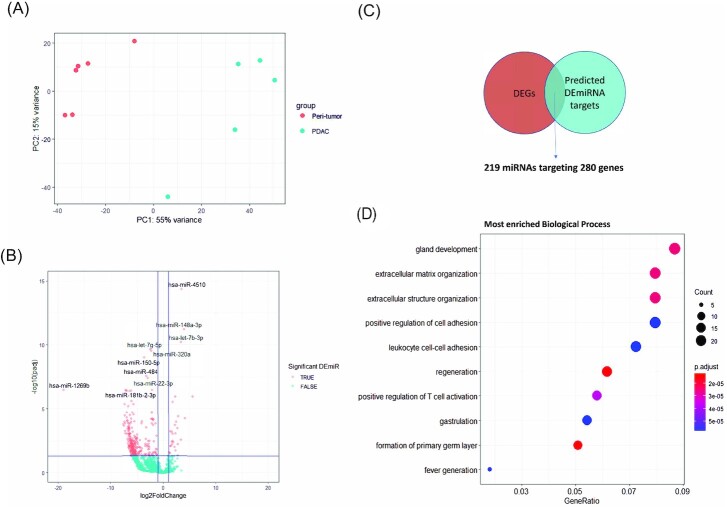
Differential circulating miRNA expression between PDAC and control subjects. (**A**) PCA plot displaying clustering of plasma miRNA expression between 6 control samples and 5 PDAC samples. (**B**) Volcano plot showing differentially expressed miRNAs in plasma between PDAC subjects and control. –log10 (padj) >1.3 (padj < 0.05) and absolute log2FoldChange > 1 are marked as the significant threshold. The top 10 significant DEmiRNAs are highlighted. (**C**) Venn Diagram showing that 280 of all the DEGs from RNA-seq data were predicated targets of 219 of plasma DEmiRNAs. (**D**) Top GO term Biological Process enrichment by plasma DEmiRNA targeted DEGs.

Considering the role of extracellular miRNAs in mediating TME reprogramming and the fact that extracellular miRNAs could be released or leaked into circulation, we carried out plasma miRNA sequencing for both PDAC patients and control participants to investigate the potential role of circulating miRNAs in PDAC TME development. An average of 3 million mapped miRNA reads were obtained from each sample. The global PCA showed clear separation between PDAC and control subjects (Fig. 4A). A total of 322 significantly differentially expressed miRNAs (DEmiRNAs) were identified (padj < 0.05 and absolute log2FoldChange > 1) (Supplementary Table S6). As shown in the volcano plot (Fig. 4B), miR-1269b, miR-181b-2, and let-7g were among the significantly down-regulated miRNAs; while miR-514a, let-7b, and let-7c were upregulated. A comparison between the significant DEmiRNAs in plasma and tumor tissue revealed 10 common DEmiRNAs (Supplementary Fig. S7A) of which three were upregulated (miR-10a-5p, miR-514a-5p, and miR-574–3p) and one was downregulated (miR-216b-5b) in both PDAC tumor tissue and plasma. miRNA-gene opposite expression pairing analysis identified 778 miRNA-gene interactions between 219 DEmiRNAs and their 280 target DEGs in PDAC plasma (Fig. 4C, Supplementary Table S7). Intersection showed that 74 genes, which accounted for 63% of all tissue DEmiRNA targets and 26% of all plasma DEmiRNA targets, were common targets of both tissue and plasma DEmiRNAs (Supplementary Fig. S7B). The GO term enrichment analysis based on the target DEGs indicated these DEmiRNAs in plasma mostly target TME processes such as ECM organization and immune responses (Fig. 4D).

**Figure 5.**
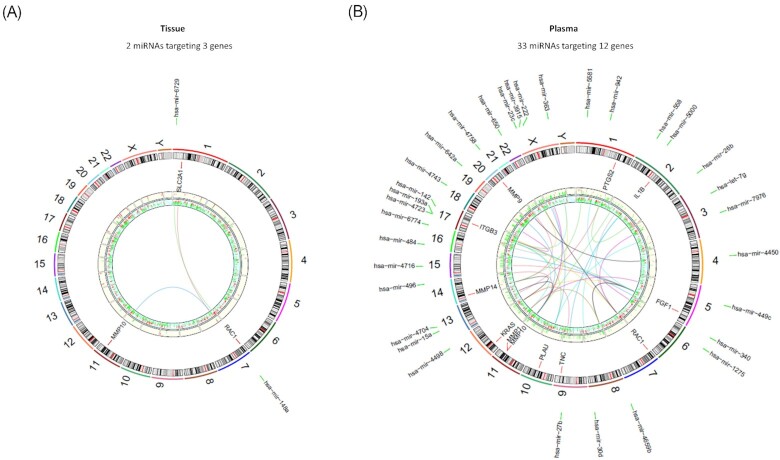
Circos plot showing DEGs, DEmiRNAs and miRNA-gene interactions involved in tumor microenvironment (TME) signaling pathways in PDAC tumor tissue (A) and plasma (B). Each circle from the periphery to the core is annotated as the following: chromosomal location in beige background; significant DEmiRNAs detected in miRNA-seq in white background; significant DEGs detected in RNA-seq, with red indicating upregulation and green indicating downregulation; and the height of the bars represents relative expression levels; in the center, miRNA-gene interactions in TME signaling. The outer circle texts annotate the DEmiRNAs and the inner circle texts annotate the DEGs targeted by the DEmiRNAs in TME signaling.

Using IPA, we searched for miRNA-gene pairs involved in TME signaling. We identified two tumor tissue miRNAs with three target genes, and 33 plasma miRNAs with 12 target genes (Supplementary Tables S4 and S7). Figure 5 illustrates the miRNA-gene interactions involved in TME signaling in tissue (A) and plasma (B), respectively. Because more miRNA-gene pairs associated with TME signaling were identified from plasma miRNAs and DEGs pairing, and plasma miRNAs are believed to be more viable as potential TME biomarkers due to its stability and easy accessibility, we focused on the potential regulatory roles of plasma miRNAs in PDAC TME reprograming. In sum, we identified multiple TME signaling pathways targeted by downregulated PDAC patient plasma miRNAs. The DEmiRNA enrichment analysis predicated up-regulation in the transcription of the TME associated genes, e.g. miRNAs targeted ECM remodeling genes such as MMP family members, *FGF1* (fibroblast growth factor 1), *PLAU* (plasminogen activator urokinase), etc. (Supplementary Fig. S1). Interestingly, functional analysis hinted that promotion in cell growth, morphogenesis, tumor growth and invasion were the most likely regulated biological processes targeted by the plasma DEmiRNAs identified in our dataset.

Previous studies have suggested the prognostic value of the immune and stromal signatures in PDAC TME.^18,19^ To explore the effectiveness of circulating miRNAs on patient prognostics, we investigated the correlation between the expression of genes targeted by circulating DEmiRNAs in TME and the predicated survival time of PDAC patients (Kaplan-Meier survival analysis, Supplementary Fig. S2). In this analysis, we found that elevated gene expression was significantly reversely correlated with the predicated survival rate in 10 of the 12 DEGs regulated by circulating DEmiRNAs (*KRAS, RAC1, PTGS2, PLAU, TNC, IL1B, MMP1, MMP9, MMP10, and MMP14*). Our results strongly support the notion of using circulating miRNAs as PDAC prognostics markers.

### scRNA-seq revealed cellular context of the TME reprogramming regulated by circulating miRNAs

**Figure 6.**
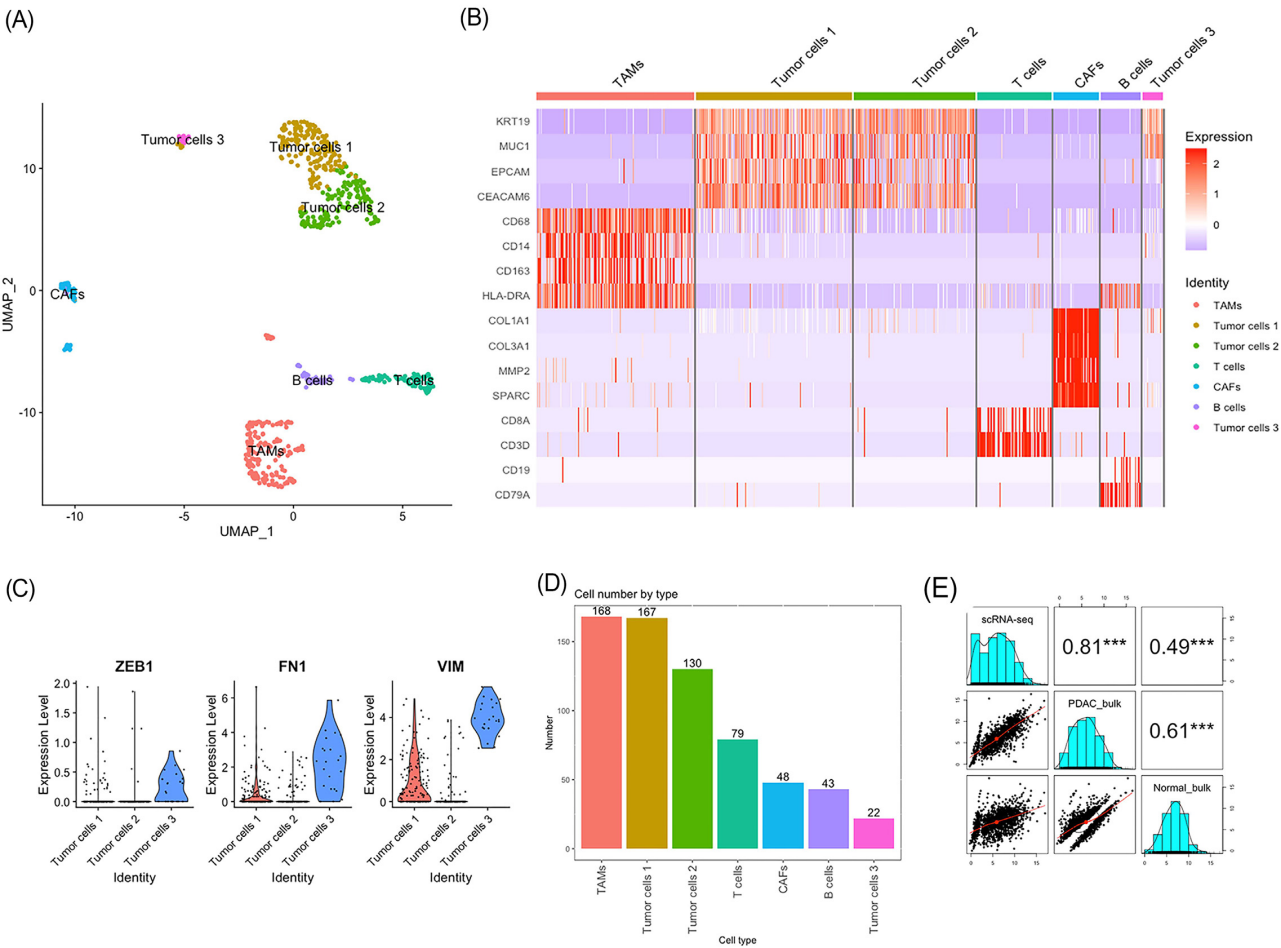
scRNA-seq of PDAC tumor tissue identified seven cell populations. (**A**) UMAP showing seven different clusters and cell types with specific cell type marker gene expression from a PDAC tissue. (**B**) Heatmap depicting the expression of known marker genes across seven different cell populations (TAMs, Tumor cells 1, Tumor cells 2, T cells, CAFs, B cells and Tumor cells 3) based on scRNA-seq data. (**C**) Violin plots showing the epithelial-mesenchymal transition signature gene expression in Tumor cells 3. (**D**) Bar plot showing the distribution of cell numbers across cell types based on scRNA-seq data. (**E**) Pearson correlation between scRNA-seq and bulk RNA-seq data.

To gain further understanding on the TME, we performed single-cell RNA-sequencing on one fresh PDAC tumor sample using 10x Genomics Chromium Single Cell 5′ Library kit. As a note, there was severe fibrosis in other tumor tissues that would not allow us to perform scRNA-seq. After data normalization, PCA, and dimension reduction using UMAP, our PDAC tumor single cells were projected into seven distinct clusters on 2-dimensional UMAP and the cell type in each cluster was identified by well-established cell type markers (Fig. 6A). The expression of the marker genes in each cell type was visualized by a heatmap shown in Fig. 6B. Out of the seven cell types identified, three were tumor cells: tumor cell population 1 (tumor cells 1), tumor cell population 2 (tumor cells 2), and tumor cell population 3 (tumor cells 3). All tumor cells showed high expression in signature genes of pancreatic epithelial lesion, like *KRT19, MUC1, EPCAM* and *CEACAM6*. It is worth noting that tumor cells 3 cluster exhibited high expression of mesenchymal markers (*ZEB1, FN1*, and *VIM*) compared to the other two tumor cell clusters (Fig. 6C), suggesting that this population of cells may have undergone epithelial-mesenchymal transition. Tumor-associated macrophages (TAMs) were identified by *CD68, CD14, CD163* and *HLA-DRA* and cancer-associated fibroblasts (CAFs) were characterized by *COL1A1, COL3A1, MMP2*, and *SPARC*. We also identified T cells by *CD8A* and *CD3D* as well as B cells by *CD19* and *CD79A* (Figs. 6A, 6B). Additional information on cell type marker expression is provided in Supplementary Fig. S3. The cell number for each population captured is displayed in Fig. 6D. To examine how well the scRNA-seq was correlated with the bulk-cell RNA-seq data, we performed Pearson correlation analysis which showed that PDAC tumor single-cell transcriptome had a high correlation with tumor bulk transcriptome (r = 0.81), but a low correlation with peri-tumor bulk-cell RNA-seq data (r = 0.49) (Fig. 6E). Furthermore, to investigate if there was high copy number variation (CNV) in the cancer cells, we performed inferred gene copy number analysis based on scRNA-seq data using R package inferCNV, then verified the cell type assignment based on average gene expression pattern across genome. Using CAFs as reference cells, we observed significantly more copy number variations across all chromosomes in three tumor cell populations, which is consistent with the notion that tumor cells harbor large number of CNVs. We also detected higher CNVs on chromosome 6 in TAMs, T cells, and B cells (Supplementary Fig. S4).

Earlier, we have identified several upregulated bulk RNA-seq DEGs which are involved in TME signaling and targeted by circulating DEmiRNAs (targets of interest described earlier). To further characterize the expression of those TME signaling genes, we visualized their expression in different cell types, using scRNA-seq (Supplementary Fig. S5). We discovered that *KRAS* was abundantly expressed in all tumor cell populations as well as in CAFs and T cells. *RAC1* was expressed in every cell type, however its expression was significantly higher in three tumor populations (Supplementary Fig. S5). *RAC1* is a target gene of miR-6729-5p, a tissue DEmiRNA. This result hinted that tumor tissue miRNA directly regulates TME-related gene expression. *PLAU* and *MMP14* were highly expressed in both tumor cells 3 and CAFs but were also consistently detected in TAMs and tumor cells 1. *TNC* was mainly expressed in CAFs, while *MMP1* was mostly expressed in tumor cells 3. *MMP9, PTGS2*, and *IL1B* were almost exclusively expressed in TAMs.

## Discussion

The roles of tumor microenvironment in cancer initiation and progression have been recognized in many studies.^4^,^20–22^ Previous studies have explored the PDAC miRNA expression profile to identify potential biomarkers for diagnosis or prognosis purpose. However, many attempts were either only focused on biomarker screening or meta-analysis from publicly available data and few were functionally or mechanistically driven. Additionally, recent studies have shown the regulatory roles of miRNAs in several tumor/stromal cell populations, in which miRNAs orchestrate the remolding of TME and tumor progression in multiple malignancies, including endometrial cancer and breast cancer.^11^ Nevertheless, studies focused on miRNA modulated TME signaling in PDAC are still lacking. In this study, we investigated the abnormal miRNA expression in PDAC tissue and patient plasma, integrated miRNA expression with cancer tissue RNA expression with a focus of interrogating TME signaling pathways modulated by miRNAs. This work helped understand the dynamics between miRNAs and TME components. Our results suggested that DEmiRNAs in PDAC plasma were mostly targeting TME signaling while tumor tissue DEmiRNAs were more associated with pancreatic functions. Given the stability and easy accessibility, our results indicated that plasma circulating miRNAs may be more promising as TME biomarkers. A few potential candidates are hsa-let-7g-3p, hsa-let-7i-3p, and hsa-miR-5000-5p. Their expressions were significantly suppressed in PDAC patient plasma (Supplementary Table S6), and their predicated targeted genes are involved in promoting TME, tumor growth and invasion (Supplementary Table S7). Further, we identified multiple TME pathways suppressed by PDAC plasma miRNAs (Supplementary Table S7). Our study not only corroborated the regulatory roles of plasma miRNAs in the TME reprogramming and PDAC progression, but discovered a sizable pool of plasma miRNA as potential PDAC biomarkers.

We identified 1445 DEGs in PDAC tumor tissue and many of those were involved in the activation of the PDAC oncogenic signature pathways such as KRAS signaling and P53 signaling. The top enriched biological processes from those DEGs included peptide secretion, extracellular matrix, and structure organization (Fig. 2). Together, our results indicated a highly active EMC remodeling process in the PDAC TME. Consistent with previous report, our results showed CAF activation plays a role in PDAC TME development. Activated CAFs, together with some tumor cells, deposit a large amount of ECM proteins and reshape the extracellular matrix.^23^

To gain a better understanding on how miRNAs regulate the ECM remodeling in PDAC, we identified several abnormally expressed miRNAs in PDAC tumor tissue (Fig. 3), some of which, like miR-216 family, miR-217, miR-135a, miR-148a, miR-891a, miR-196a, miR196b, miR-222, miR-375, and miR-29c, were also reported in other studies.^16,24,25^ We found there were more miRNAs with altered expression in PDAC plasma than in the PDAC tumor tissue (Fig. 4). Specifically, there was a decreased expression of certain known tumor suppressor miRNAs such as miR-216–5p and miR-126-3p in the plasma.^26,27^ Some members of the let-7 family, including let-7g, let-7i, let-7a, and let-7f were also decreased in PDAC plasma. The altered expressions of let-7a and let-7f were consistent with the findings by Nweke and Brand.^28^ Interestingly, miR-214-5p, a miRNA whose low expression was associated with CAF activation in ovarian cancer,^29^ was significantly decreased in PDAC plasma. In our study, there were only ten overlapping (common) DEmiRNAs between tumor tissue and plasma (Supplementary Fig. S6). Similar observations were reported by other studies as well.^17^ One explanation is that some circulating miRNAs were selectively secreted by either tumor tissue for tumor promotion or by immune cells in response to the tumor invasion, in either case resulting in different DEmiRNA profiles between tumor tissue and plasma. Two distinct miRNA target profiles between tumor tissue and plasma were discovered by GO term enrichment analysis based on DEmiRNAs, i.e., pancreatic secreting functions were associated with tumor tissue DEmiRNAs, while TME reprogramming functions, especially ECM remodeling, were associated with plasma DEmiRNAs.

We further discovered that the increased expression of multiple TME genes targeted by plasma DEmiRNAs were associated with worse clinical outcomes, which emphasized the role of plasma miRNAs on PDAC tumor promotion by regulating TME (Supplementary Fig. S2). In particular, MMP family members (*MMP1, MMP9, MMP10, MMP14*), important players in TME remodeling and the targets of miRNAs, were suppressed in PDAC plasma. Other miRNAs targets, like *PLAU* (plasminogen activator, urokinase) participating in ECM (involved in tissue remodeling and tumor migration^30^), were also suppressed in PDAC plasma. miRNAs targeting *IL1B*, a cytokine promoting immune suppression and tumor proliferation through tumor stroma interaction,^31^ were downregulated in PDAC plasma as well. Similar suppression of TME regulating miRNAs were also observed in tumor tissue, albeit with only three interactions identified (Fig. 5). The close interplay between plasma miRNA and TME signaling strengthens the notion that circulating miRNAs may serve as good biomarkers for PDAC and further led us to examine the role of dysregulated plasma miRNAs in PDAC TME signaling in great detail.

In recent years, miRNAs, especially circulating miRNAs, have gained recognition as crucial mediators in facilitating tumor-stromal communication by shuttling between various types of cells via membrane-derived vehicle (*e.g*., exosome).^32^ It is known that miRNAs are exported from cancer cells (CAFs, TAMs, and other immune cells in TME) to orchestrate tumor growth and drug resistance.^33^ In attempt to shine some light onto the role of miRNA in modulating TME signaling in various PDAC TME components, we conducted single-cell RNA-seq on PDAC patient tumor sample. We identified three tumor cell populations (including one that had undergone EMT), TAMs, CAFs, T cells, and B cells based on the gene expression of established marker genes (Fig. 6B). The tumor cell characteristics was also confirmed by the inferCNV results, with high CNV density across all chromosomes in three tumor cell populations, consistent with reports of the profound aneuploidy of the malignant ductal cells.^16^ We also noticed that in T cells, B cells, and TAMs, higher copy number variations were found on chromosome 6 which harbors numerous genes involved in immune responses. This is consistent with the characteristics of immune cells infiltrated into the cancer tissue. Given the significant differences among malignant ductal cell subpopulations from different PADC patients,^34,35^ we acknowledge the limitation of the characterization on the three tumor populations from a single patient. In future study, scRNA-seq from large cohort with sufficient normal pancreas ductal cells are necessary. This will make direct comparison between malignant and normal ductal cells possible, ensuring better understanding of the heterogeneity of TME.

We further evaluated the expressions of TME associated genes targeted by (or paired with) plasma DEmiRNAs (genes of interest, defined earlier) in different cell populations (Supplementary Fig. S5). For intracellular molecules, there were significant expression of *KRAS* in all three tumor cell populations as well as in CAFs and T cells. *KRAS*, the driver mutation for PDAC, was known to affect various TME components including CAFs, immune cells, and extracellular matrix, to create a favorable TME for tumor growth by providing metabolic support, stimulating angiogenesis, and suppressing immune surveillance.^36,37^ Our findings suggested that miRNAs play a pivotal role in the cascading effect of *KRAS* induced tumor promotion. In addition, we found that the expression of *RAC1*, which encodes an important signaling molecule downstream of *KRAS* pathway, was significantly higher in all three tumor populations (Supplementary Fig. 5). This was consistent with the marked reduction of its regulatory miRNA-6729-5p in tumor tissue (Supplementary Table S3). *RAC1* plays a key role in remodeling the actin cytoskeleton and regulation of cell motility and migration, as well as in cell survival and proliferation.^38^ Our result clearly revealed that DEmiRNAs directly regulated the expressions of TME-related genes at single-cell level in tumor tissue.

We found that several miRNA-targeted genes were highly expressed in the EMC of CAFs including *MMP14, PLAU*, and *TNC*. As mentioned earlier, *MMP14* and *PLAU* both encode proteases that degrade ECM and play important roles in EMC reorganization and remodeling.^39,40^ TNC (tenascin-C), an ECM glycoprotein which binds to integrin receptors or other EMC proteins, promotes tumor invasion by influencing cell mobility and adhesion.^41^ In accordance with previous findings that miRNA mediated regulation on CAFs in multiple malignancies,^27,36,37^ our results indicated that inhibition of genes targeted by miRNAs in TME potentially promotes ECM remodeling through CAFs and facilitates cell-ECM interactions, which in turns enhance TME remodeling and tumor progression in PDAC.

Interestingly, several extracellular matrix protein genes predominantly expressed in CAFs, such as *PLAU, MMP14*, were also highly expressed in the mesenchymal-like tumor population (Tumor cells 3). Growing evidences have demonstrated the involvement of CAFs and ECMs in PDAC tumor cells epithelial-mesenchymal transition (EMT),^42^ as it has been suggested that CAFs drove EMT in PDAC by secreting various cytokines such as TGF-β, IL-6.^42,43^ In addition to providing a hypoxic environment, ECM molecules also mediate signaling from tumor cells to engage EMT pathways.^23,42^ Additionally, another target gene *ITGB3* (integrin beta 3), a cell surface receptor facilitating cell-EMC interaction, was primarily expressed in the Tumor cells 3 population. These findings highlighted the possibility that CAFs and ECM drive EMT through miRNA mediated networks.

We also found *IL1B*, a cytokine promoting immune suppression in TME,^31^ was primarily expressed in TAMs, which functions like PTGS2 (cyclooxygenase-2), a key enzyme contributing to TAM polarization and immune escape.^44^ These findings suggested the contribution of miRNAs in the immunosuppression within PDAC TME facilitated by TAMs.

We acknowledge there are limitations of our study. Our sample size was small which may have affected the statistical power and differentially expressed genes or miRNAs identified, considering the potential biological and technical variations. Also, due to the severe fibrosis of the pancreatic cancer tissues, scRNA-seq data was obtained from only one patient tumor sample. However, the aim of this study was to explore the potential role of miRNAs within TME dynamics. To improve the reliability of our study in our single-cell study, we only studied those miRNA-targeted genes whose increased expression was significantly associated with PDAC patient survival because we focused more on the potential prognostic values of the miRNAs targeted genes. Nevertheless, we acknowledge that further functional validations are required to dissect the underlying mechanism of miRNA regulatory networks in TME.

## Conclusion

Using patient PDAC tumor and adjacent peri-tumoral tissue samples, we discovered that EMC remodeling was one of the most enriched pathways in PDAC progression. We identified 322 and 49 abnormally expressed miRNAs in PDAC patient plasma and tumor tissue, respectively. Paired miRNA-gene analysis suggested that differentially expressed miRNAs in plasma mainly target various TME signaling pathways, indicating the potential of plasma miRNAs as biomarkers for PDAC TME. Integrating scRNA-seq with miRNA-seq profiling, we found a multi-faceted role of plasma miRNAs in PDAC TME remodeling. Through the suppression of miRNAs targeting various TME signaling, tumor cells together with CAFs and TAMs orchestrated *KRAS* induced tumor promotion, ECM remodeling, epithelial-mesenchymal transition as well as immunosuppression in PDAC TME, which favors tumor promotion. In summary, our study highlighted the important role of miRNAs, as mediators that were heavily involved in TME reprogramming and heterogeneity. The inhibition of circulating miRNAs targeting these key TME pathways in the stromal components may present these miRNAs as promising biomarkers for pancreatic cancer therapy and for assessing the PDAC TME characteristics and functions.

## Supplementary Material

pbad004_Supplemental_Figures

## Data Availability

The sequencing data have been deposited to the NCBI GEO (Gene Expression Omnibus) under the GEO Submission number GSE226762. We used many publicly available algorithms and packages for the miRNA-seq, RNA-seq and scRNA-seq mapping, genome annotation, differential analysis, etc., which were cited properly in the paper. This paper did not produce original code.
