# Supplementary material for: Dysregulated miRNAs modulate tumor microenvironment associated signaling networks in pancreatic ductal adenocarcinoma
Source: Precis Clin Med. 2023 Mar 10;6(1):pbad004. doi: 10.1093/pcmedi/pbad004 (PMC10052370; doi:10.1093/pcmedi/pbad004)
Supplement: pbad004_Supplemental_Figures [file pbad004_supplemental_figures.zip › Supplementary_Figures_03022023.pdf]

Dysregulated miRNAs modulate tumor microenvironment associated signaling networks in pancreatic ductal adenocarcinoma

Tiantian Liu[1,*], Zhong Chen[1,*], Wanqiu Chen[1], Ryan Evans[2], Jane Xu[1], Mark E. Reeves[3], Michael de Vera[2], Charles Wang[1,4#]

Supplementary Figures

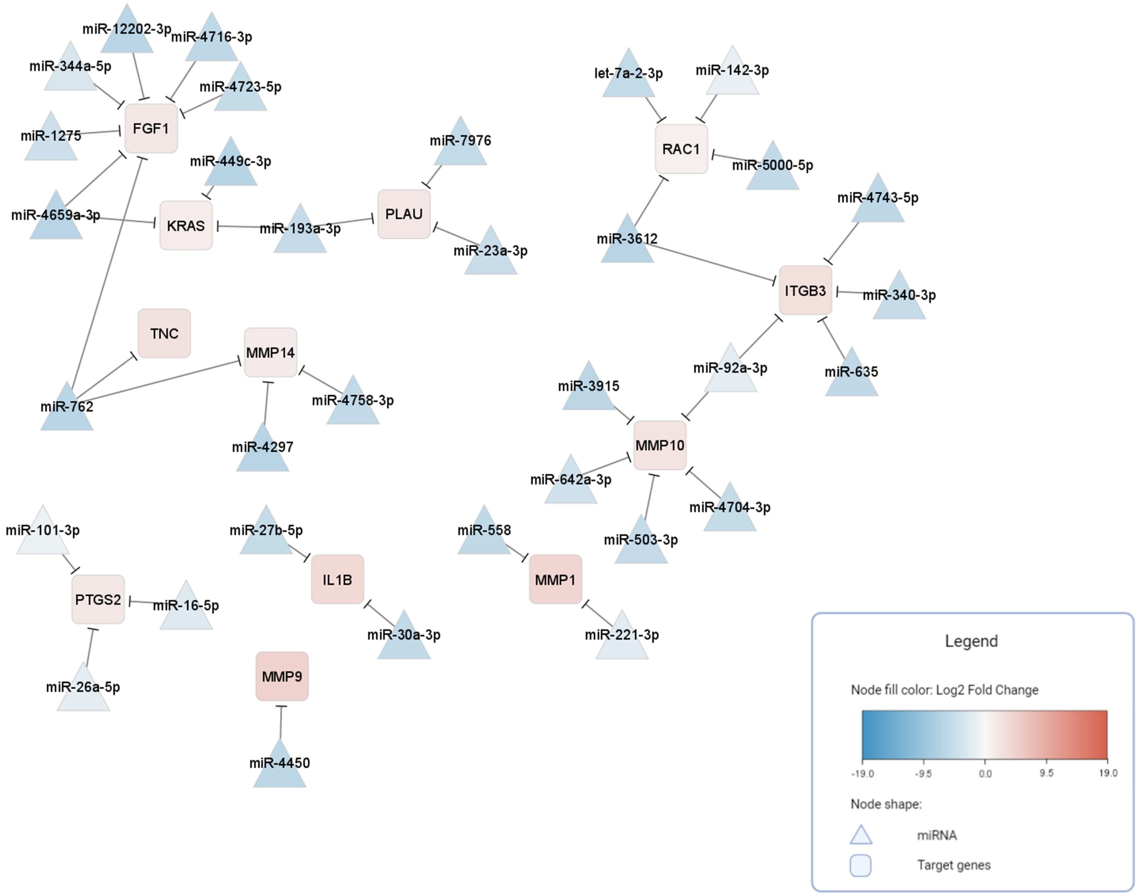

Supplementary Figure S1. Interaction network of DEmiRNAs and their target genes in PDAC TME signaling. Color scale is in increasing order of Log2 Fold Change from blue to red. Triangles represent miRNAs and rectangles represent genes.

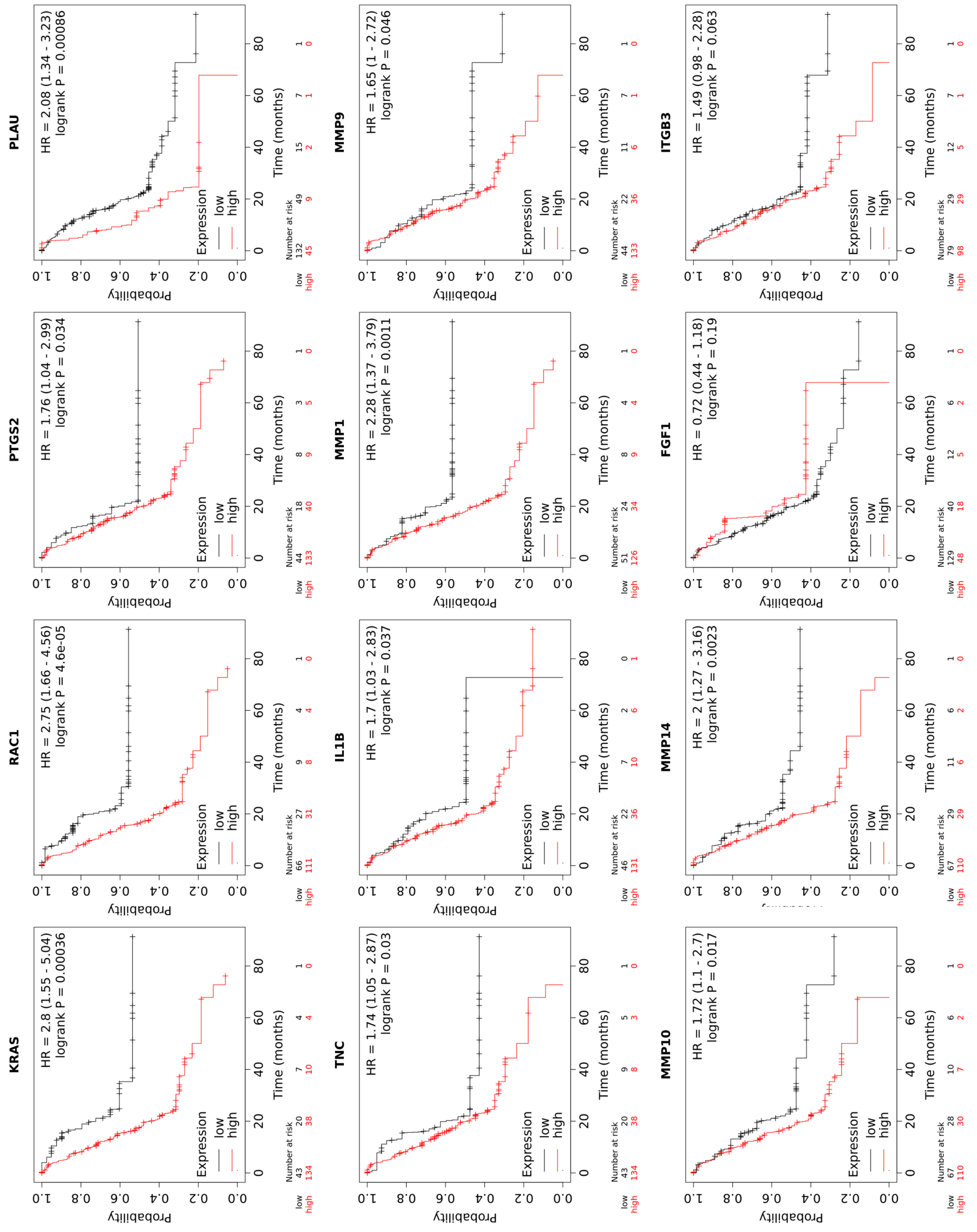

Supplementary Figure S2. Kaplan-Meier curves showing the association of the overall PDAC patients survival time and the expression of TME signaling genes regulated by PDAC circulating DE miRNAs. The plots were generated using KM plotter.

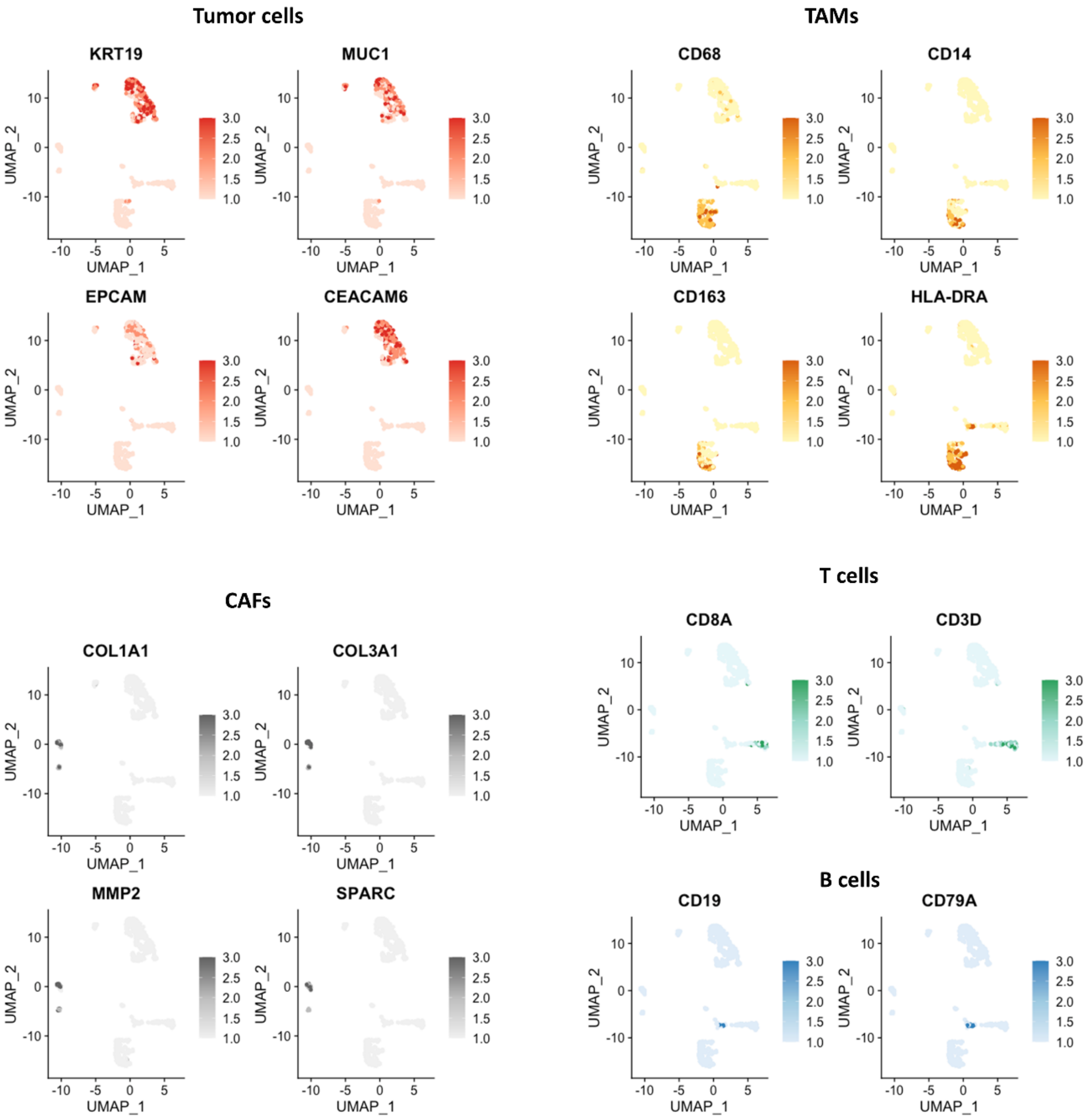

Supplementary Figure S3. Feature plots showing known markers expressed in different cell populations (Tumor cells, TAMs, CAFs, T cells and B cells).

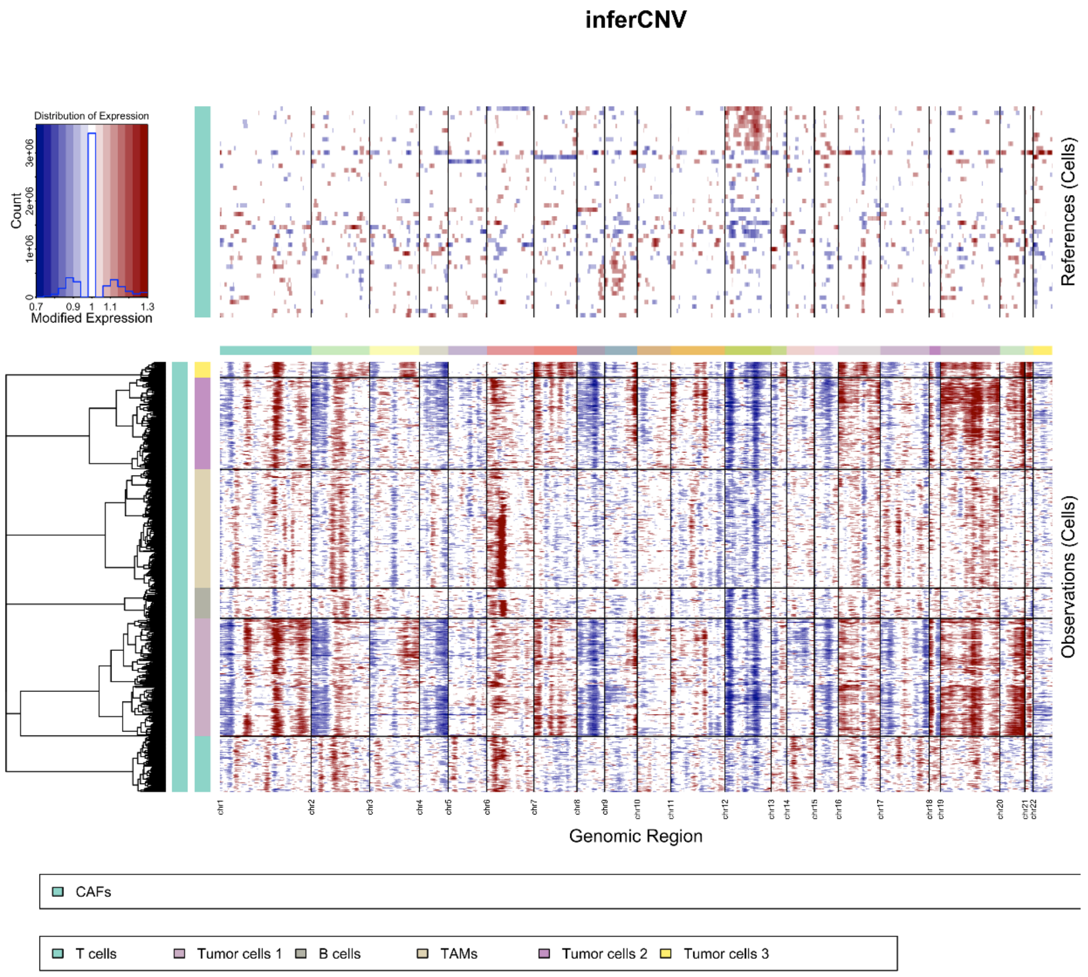

Supplementary Figure S4. Inferred copy number variation (CNV) analysis in 7 different cell types. CAFs demonstrated relatively uniformed copy numbers across all chromosomes and was used as reference for copy number comparison. Cell types were indicated by color bars on the left of heatmap. Three tumor cell populations showed high CNVs across multiple chromosomes. TAMs, T cells and B cells showed high CNVs on chromosome 6.

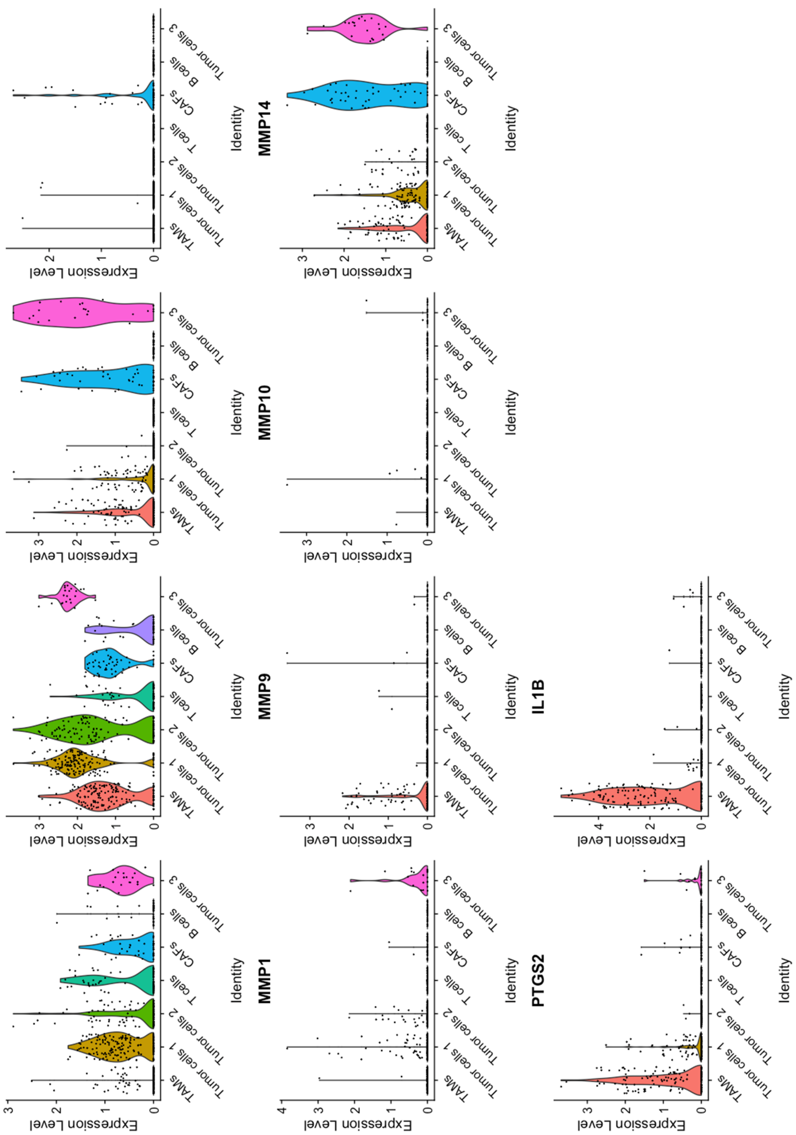

Supplementary Figure S5. Violin plots showing the gene expression of TME signaling associated genes targeted by PDAC circulating DE miRNAs in 7 cell populations. Y-axis shows the specific up-regulated target DEGs involved in TME signaling. X-axis shows cell type identify based on scRNA-seq data.

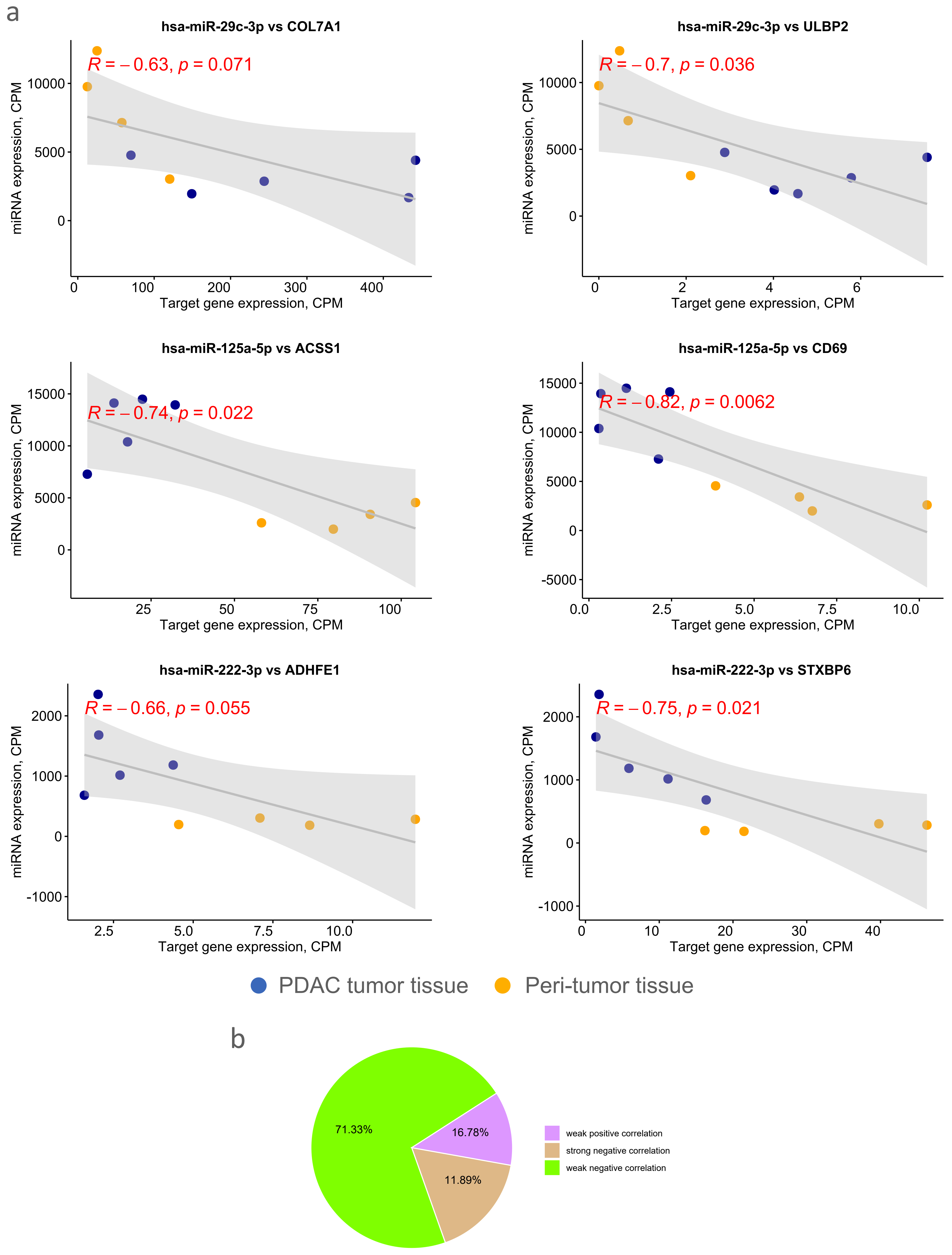

Supplementary Figure S6. **Co-expression analysis of PDAC tumor DE-miRNAs and their target genes**. **a**) Pearson correlation plots of the expressions of representative miRNA-target gene pairs; **b**) Summary of co-expression of 143 miRNA-target gene pairs. Strong correlation was defined as |R|>0.6 and p <0.1.

a

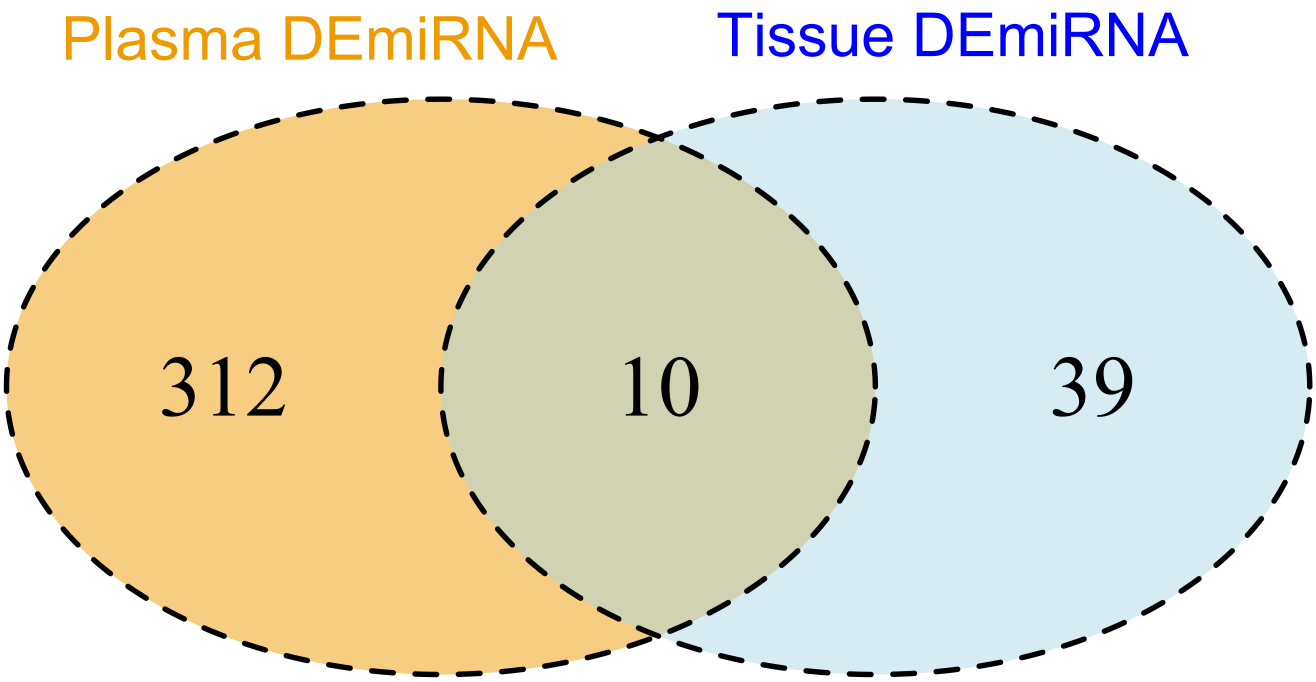

b

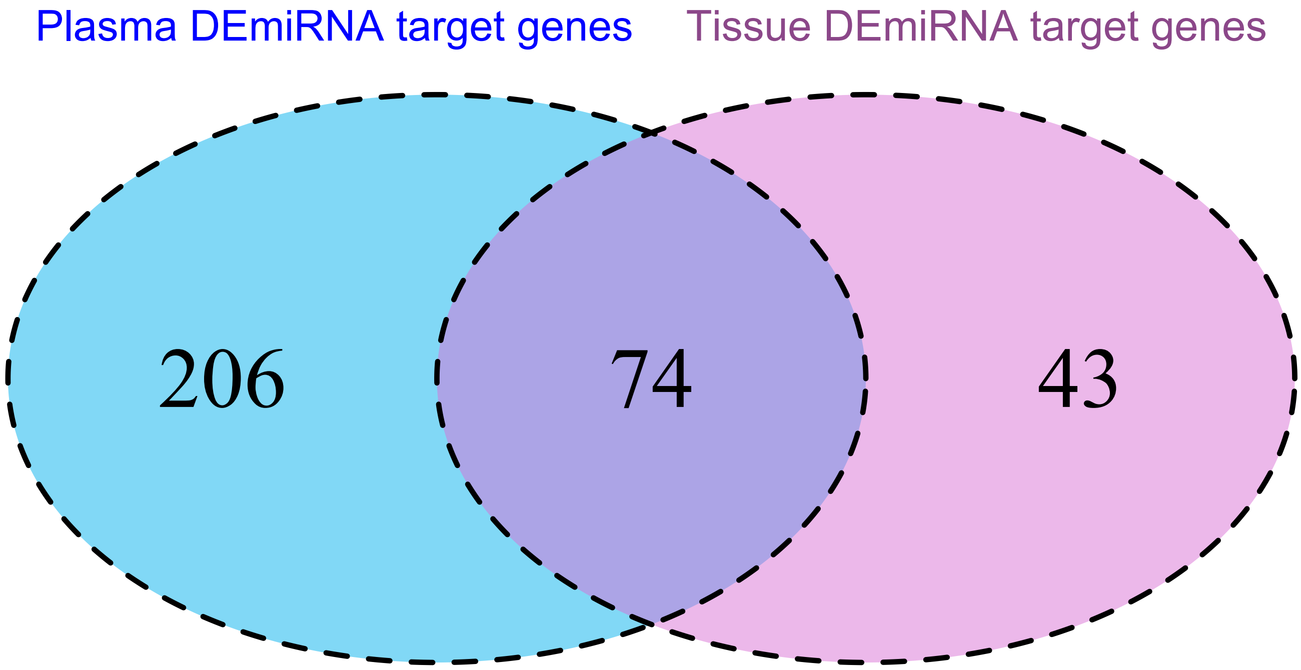

Supplementary Figure S7. Venn diagrams showing overlapped DEmiRNAs and DEmiRNA-target genes between plasma and tissue. **a**) The numbers of differentially expressed miRNAs (DEmiRNA) in PDAC patient plasma and PDAC tumor tissue, as well as the common DEmiRNAs; **b**) the numbers of differentially expressed genes (DEGs) identified in PDAC tumor tissue that were targets of DEmiRNAs.
